# Accelerometer-measured physical activity is associated with knee breadth in middle-aged Finns – a population-based study

**DOI:** 10.1186/s12891-022-05475-7

**Published:** 2022-05-31

**Authors:** Juho-Antti Junno, Asla Keisu, Maisa Niemelä, Marella Modarress Julin, Raija Korpelainen, Timo Jämsä, Jaakko Niinimäki, Petri Lehenkari, Petteri Oura

**Affiliations:** 1 Medical Research Center, Oulu University Hospital and University of Oulu, Oulu, Finland; 2 Cancer and Translational Medicine Research Unit, Faculty of Medicine, University of Oulu, Oulu, Finland; 3 Department of Archaeology, Faculty of Humanities, University of Oulu, Oulu, Finland; 4 Archaeology, Faculty of Arts, University of Helsinki, Helsinki, Finland; 5 Research Unit of Medical Imaging, Physics and Technology, University of Oulu, Oulu, Finland; 6 Center for Life Course Health Research, Faculty of Medicine, University of Oulu, Oulu, Finland; 7 Department of Sports and Exercise Medicine, Oulu Deaconess Institute Foundation sr., Oulu, Finland; 8 Department of Forensic Medicine, Faculty of Medicine, University of Helsinki, Helsinki, Finland; 9 Forensic Medicine Unit, Finnish Institute for Health and Welfare, Helsinki, Finland

**Keywords:** Knee, Anthropometry, Radiography, Accelerometer, Epidemiology, Finland

## Abstract

**Background:**

Articular surface size is traditionally considered to be a relatively stable trait throughout adulthood. Increased joint size reduces bone and cartilage tissue strains. Although physical activity (PA) has a clear association with diaphyseal morphology, the association between PA and articular surface size is yet to be confirmed. This cross-sectional study aimed to clarify the role of moderate-to-vigorous PA (MVPA) in knee morphology in terms of tibiofemoral joint size.

**Methods:**

A sample of 1508 individuals from the population-based Northern Finland Birth Cohort 1966 was used. At the age of 46, wrist-worn accelerometers were used to monitor MVPA (≥3.5 METs) during a period of two weeks, and knee radiographs were used to obtain three knee breadth measurements (femoral biepicondylar breadth, mediolateral breadth of femoral condyles, mediolateral breadth of the tibial plateau). The association between MVPA and knee breadth was analyzed using general linear models with adjustments for body mass index, smoking, education years, and accelerometer weartime.

**Results:**

Of the sample, 54.8% were women. Most individuals were non-smokers (54.6%) and had 9—12 years of education (69.6%). Mean body mass index was 26.2 (standard deviation 4.3) kg/m^2^. MVPA was uniformly associated with all three knee breadth measurements among both women and men. For each 60 minutes/day of MVPA, the knee breadth dimensions were 1.8—2.0% (or 1.26—1.42 mm) larger among women (*p* < 0.001) and 1.4—1.6% (or 1.21—1.28 mm) larger among men (*p* < 0.001).

**Conclusions:**

Higher MVPA is associated with larger tibiofemoral joint size. Our findings indicate that MVPA could potentially increase knee dimensions through similar biomechanical mechanisms it affects diaphyseal morphology, thus offering a potential target in reducing tissue strains and preventing knee problems. Further studies are needed to confirm and investigate the association between articulation area and musculoskeletal health.

**Supplementary Information:**

The online version contains supplementary material available at 10.1186/s12891-022-05475-7.

## Introduction

Physical activity (PA) and skeletal loading affect bone strength primarily via bone mineral density, mineral content and geometric properties [1–3]. The importance of PA for skeletal health is widely recognized, but a clear consensus about the optimal type and level of PA for skeletal health is still lacking. In our previous work we showed that objectively measured physical activity was associated with increased vertebral size in the lumbar spine [4].

In light of the connection between PA and bone geometry [5], weight-bearing joints could be assumed to be adaptable like long bone shafts [6]. This would be important as increased articular surface size reduces bone and cartilage tissue strains [7]. However, factors that are associated with articular surface dimensions are not completely agreed upon, and articular surface size is commonly understood to remain relatively stable throughout adulthood. Major lifetime factors such as physical activity seem to have a minimal effect on joint size [8–10]. Ruff and colleagues [8] concluded that the femoral head seems to be a very conservative structure with minimal response to changes in mechanical loads. It is hypothesized that the acetabulum restricts the potential growth of the proximal head of femur but studies with mice have also found that femoral head can adapt to exercise (e.g. [6]). Lieberman and colleagues [9] concluded in their study with sheep that diaphyseal cross-sectional geometry is an appropriate proxy for activity level, but articular surface area has no response mechanism for mechanical loadings.

In this population-based study, we wanted to clarify the potential association between PA and knee joint dimensions in humans. We used a general population sample of 1508 Northern Finnish women and men, with accelerometric data on PA and knee radiographs at the age of 46. We focused on accelerometric PA data as over- or underestimation of absolute PA levels is a common problem in self-reporting [11, 12]. Based on previous findings on lumbar vertebrae [4], we hypothesized that higher levels of moderate-to-vigorous PA (MVPA) would also be associated with larger knee breadth, thus implying reduced bone and cartilage tissue strains.

## Methods

### Study sample

Northern Finland Birth Cohort 1966 (NFBC1966) is a prospective, population-based birth cohort started in 1960s. At baseline all mothers with expected dates of delivery between the 1st of January and the 31st of December 1966 were recruited from the two northernmost provinces in Finland. NFBC1966 population comprised 12,231 children (12,058 live births) and their parents. Follow-ups have been conducted at several time points. In adulthood, data have been collected for example on social background, lifestyle, and organ-specific symptoms. Detailed descriptions can be found at [13, 14].

**Fig. 1:**
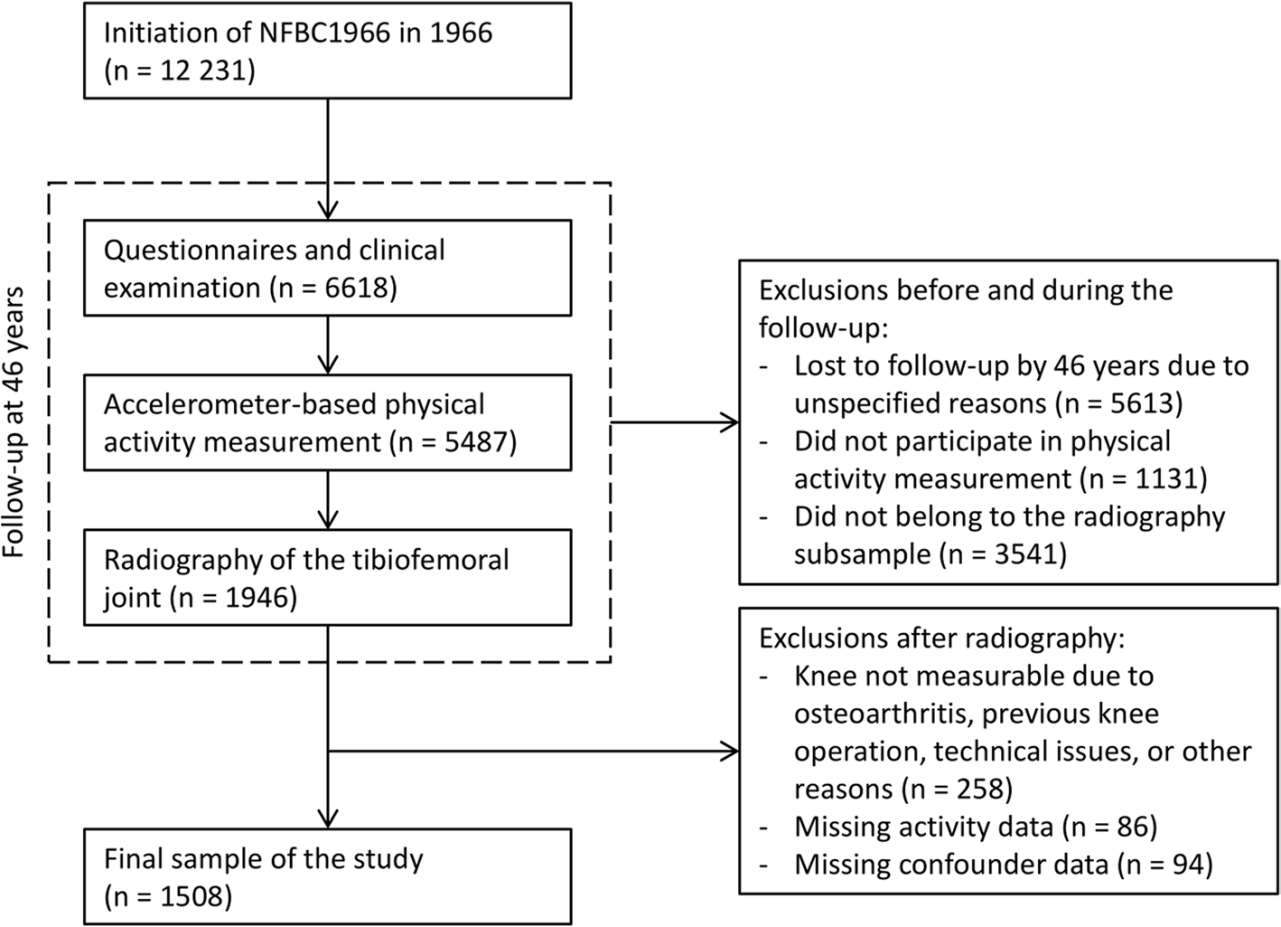
Flow-chart of the study. NFBC1966 = Northern Finland Birth Cohort 1966

To conduct this study, we examined knee radiographs, accelerometer data, and anthropometric, lifestyle and sociodemographic data from the NFBC1966 members who had participated in the 46-year follow-up. Of all individuals who participated in the follow-up, those residing in the Oulu region (100 km radius) were further invited to knee radiography. A flowchart demonstrating the formation of the sample with exclusions is presented in Fig. 1. The final sample size of our study was 1508.

### Accelerometer-based physical activity

PA was measured by a waterproof wrist-worn accelerometer, Polar Active (Polar Electro, Kempele, Finland). Accelerometers were blinded, showing only the time of day, so that participants did not get feedback of their activity during the measurement period. A detailed description of the methodology and validity of the device has been given previously [15, 16]. Briefly, Polar Active has been shown to correlate with double labelled water technique in evaluating energy expenditure during daily living (*r* = 0.88) [17]. Participants of the study were instructed to carry the accelerometer on their non-dominant wrist for at least 14 days, 24 hours per day. Our analysis included participants with at least four valid days of data recorded. A valid day was determined as at least 600 minutes/day monitoring time during waking hours. Daily averages (minutes/day) at five activity levels (very light: 1–2 MET, light: 2–3.5 MET, moderate: 3.5–5 MET, vigorous: 5–8 MET and very vigorous: ≥ 8 MET) were calculated on the basis of the threshold values used by the manufacturer [18]. In this study, we focused on MVPA (≥ 3.5 MET) [19] because it is a frequently and globally used activity level in PA recommendations [20–23]. The 3.5 MET cutpoint was selected as it showed higher agreement with ActiGraph (model GT3X) using Freedson cutpoints than Polar Active with 3 MET cutpoint [24].

### Knee breadth measurements

Knee breadth was measured from digital radiographs of the right knee joint by an author of the study (A.K.). A detailed description of the procedure has been given in a previous publication [25]. In brief, we utilized posteroanterior radiographs with individuals positioned in fixed flexion view [26, 27]. Radiographs were accessed and measurements were taken using neaView Radiology software version 2.31 (Neagen Oy, Oulu, Finland). Measurements were recorded to the nearest 0.1 mm. The initial measurements were converted into true sizes with the help of a metal calibration disc of 30 mm in diameter attached on the participant’s right leg.

The following three measurements were taken from each radiograph [25]:

1. femoral biepicondylar breadth (FBEB),
2. mediolateral breadth of the articular surface of the femoral condyles (FCML), and
3. mediolateral breadth of the tibial plateau articular surface (TPML).

FBEB was measured as the maximum breadth between the medial and lateral femoral epicondyles. FCML was measured by drawing a line tangential to the most inferior points of the femoral condyles; this line was transposed to the widest part between the femoral condyles. TPML was measured as close to the border of the tibial plateau as possible.

To evaluate the intraobserver reliability of the measurements, a total of 20 radiographs (on average every 100th image of the first round) were measured a second time. Absolute and relative technical measurement errors were calculated as previously described [25]. In brief, the intraobserver reliability of the measurements was found to be high, with absolute technical measurement errors 0.1—0.5 mm and relative errors 0.1—0.6%.

### Confounders

Body mass index (BMI), smoking, education years and accelerometer weartime were assessed as potential confounders. At the age of 46, a study nurse measured the height and weight of each participant, allowing the calculation of BMI (kg/m^2^). Smoking status was enquired using two questions (“Have you ever smoked cigarettes (yes/no)?” and “Do you currently smoke (yes/no)?”); individuals were subsequently categorized as 1) non-smokers, 2) former smokers, and 3) current smokers. Socioeconomic status was proxied via education years by asking the number of years spent at school (< 9 years, 9–12 years, > 12 years). Accelerometer weartime was defined as the average monitoring time (minutes/day) over the measurement period.

### Statistical analysis

The data were analyzed using SPSS version 27 (IBM, Armonk, NY) and Stata/MP version 17 (StataCorp, College Station, TX). The level of statistical significance was set at *P* = 0.05.

Descriptive statistics were calculated as means and standard deviations (SDs) for continuous variables with normal distributions, as medians and interquartile ranges (IQRs) for continuous variables with skewed distributions, and as frequencies and percentages for categorical variables. In order to evaluate potential selection bias, the final sample was compared to those excluded by means of independent-samples T test, Mann-Whitney U test, and Chi square test.

The associations between the primary predictor (MVPA, minutes/day) and the outcomes (FBEB/FCML/TPML, mm) were analyzed using general linear models with and without adjustments for BMI (kg/m^2^), smoking history (categorized as described above), education years (categorized as described above), and accelerometer weartime (minutes/day). The confounder candidates showed significant univariate associations with the outcomes and/or primary predictor and were therefore included as covariates in the final models. Sex stratification was used due to great discrepancy in skeletal size between men and women [28]. Beta estimates, their 95% confidence intervals (CIs), and the corresponding *P* values were extracted from the data output. In order to justify linear modelling, potential non-linear relationships were ruled out by means of restricted cubic spline regression models with three evenly spaced knots (Supplementary Fig. 1).

### Ethical approval

The study conformed to the Declaration of Helsinki and received approval from the Ethical Committee of the Northern Ostrobothnia Hospital District, Oulu, Finland (94/2011). NFBC1966 members gave informed consent for participation, and personal details were pseudonymized by identification codes. This provided full anonymity for the participants.

## Results

The study sample comprised 827 women (54.8%) and 681 men (45.2%; Table 1). The majority were non-smokers (54.6%), while a minority were current smokers (16.9%). Most individuals had 9—12 years of education (69.6%). Mean body mass index was 26.2 (SD 4.3) kg/m^2^. All three knee breadth measurements were larger among men than women (FBEB 10.1 mm, FCML 10.6 mm, and TPML 9.6 mm larger). Women had a median of 56 (IQR 39—76) minutes and men 72 (IQR 52—94) minutes of MVPA per day during the accelerometer monitoring period. The present sample included a lower percentage of current smokers than the rest of the cohort (16.9% versus 23.2%, *p* < 0.001); there were also slight differences in BMI and accelerometer weartime (Supplementary Table 1).

**Table 1:** General characteristics of the sample

Characteristic	Women	Men	All
N (%)	827 (54.8)	681 (45.2)	1508 (100)
Body mass index (kg/m^2^), mean (SD)	25.6 (4.4)	27.0 (4.0)	26.2 (4.3)
Smoking			
Non-smoker, % (n)	58.4 (483)	50.1 (341)	54.6 (824)
Former smoker, % (n)	25.0 (207)	32.6 (222)	28.4 (429)
Current smoker, % (n)	16.6 (137)	17.3 (118)	16.9 (255)
Education years			
< 9, % (n)	2.7 (22)	3.8 (26)	3.2 (48)
9—12, % (n)	69.2 (572)	70.2 (478)	69.6 (1050)
> 12, % (n)	28.2 (233)	26.0 (177)	27.2 (410)
Knee breadth dimensions			
Femoral biepicondylar breadth (mm), mean (SD)	77.8 (3.6)	87.9 (3.8)	82.4 (6.3)
Femoral condylar mediolateral breadth (mm), mean (SD)	71.7 (3.3)	82.3 (3.6)	76.5 (6.3)
Tibial plateau mediolateral breadth (mm), mean (SD)	70.3 (3.1)	79.9 (3.4)	74.6 (5.8)
Accelerometer data			
Moderate-to-vigorous physical activity (minutes/day), median (IQR)	56 (39—76)	72 (52—94)	63 (45—86)
Accelerometer wear-time (minutes/day), median (IQR)	971 (933—1005)	987 (944—1023)	977 (937—1014)

*IQR* Interquartile range, *SD* Standard deviation

Table 2 demonstrates the contribution of 60 minutes/day of MVPA to each of the three knee breadth measurements according to the unadjusted and fully adjusted models. Supplementary Table 2 shows intermediate models, with adjustments added one by one. In all models, MVPA was uniformly and positively associated with FBEB, FCML and TPML among both women and men. According to the fully adjusted models, 60 minutes/day of MVPA was associated with 1.8—2.0% (or 1.26—1.42 mm) larger knee joint among women (*p* < 0.001) and 1.4—1.6% (or 1.21—1.28 mm) larger knee joint among men (*p* < 0.001).

**Table 2:** Results from general linear models for the association between moderate-to-vigorous physical activity and knee breadth

Outcome and model	Women		Men	
	Beta^a^ (95% CI)	*P* value	Beta^a^ (95% CI)	*P* value
Femoral biepicondylar breadth (mm)				
Unadjusted model	1.00 (0.52—1.48)	**< 0.001**	0.99 (0.51—1.46)	**< 0.001**
Fully adjusted model^b^	1.42 (0.92—1.92)	**< 0.001**	1.27 (0.78—1.76)	**< 0.001**
Femoral condylar mediolateral breadth (mm)				
Unadjusted model	1.16 (0.72—1.61)	**< 0.001**	1.06 (0.61—1.50)	**< 0.001**
Fully adjusted model^b^	1.40 (0.94—1.86)	**< 0.001**	1.28 (0.82—1.73)	**< 0.001**
Tibial plateau mediolateral breadth (mm)				
Unadjusted model	1.01 (0.60—1.43)	**< 0.001**	1.03 (0.61—1.44)	**< 0.001**
Fully adjusted model^b^	1.26 (0.83—1.69)	**< 0.001**	1.21 (0.78—1.65)	**< 0.001**

*CI* Confidence interval

^a^Beta coefficients are interpreted as the contribution of 60 minutes/day of moderate-to-vigorous physical activity to the respective knee breadth measurement in mm

^b^Adjusted for body mass index, smoking, education years and accelerometer weartime

## Discussion

This population-based study showed for the first time a uniform positive association between accelerometer-based physical activity and three articular measurements from the knee joint among both women and men. Although the study was cross-sectional, our findings suggest that articular surface size may differ between activity levels, thus contradicting previous literature. We speculate that higher MVPA may improve the robusticity of the bony knee structures.

Major lifetime factors such as physical activity have been considered to have a minimal effect on joint size [8–10]. However, previous research has mainly concentrated on animal models and/or proximal femur with relatively small sample sizes. The present findings challenge these viewpoints. In light of the connection between PA and bone geometry [5], weight-bearing joints could be assumed to be adaptable like long bone shafts [6]. It has been suggested that also exercise and mechanical loading may affect articular surface size and shape (e.g. [6]). In our previous study, we were able to show a positive association between MVPA and vertebral size in the lumbar spine [4]. There thus seems to be increasing evidence that PA may affect bone size in both the axial and appendicular skeleton.

The main strength of this study is the large sample size of 1508 individuals. Our sample originated from the population-based NFBC1966 study which provides the best available proxy of the general Northern Finnish middle-aged population [13]. Additionally, the individuals in our sample were coeval which minimized the confounding effect of secular trends on our results. We were able to exclude individuals with a history of knee operations or knee pathologies such as osteoarthritis, and accounted for several potential confounders in the statistical analysis. The knee breadth measurements had high intraobserver reliability and low measurement errors. A wrist-worn accelerometer provided a reliable record of individuals’ daily total physical activity, including both occupational and leisure-time PA. Accelerometers are sensitive and practical tools for measuring PA, and arguably more reliable than self-reported PA [11, 12].

Our study has several limitations. Comparison between the present sample and those excluded showed mild selection bias, as the sample included a lower percentage of current smokers than the rest of the cohort. However, the differences were minor and have been documented previously [29]. Although the NFBC1966 has collected a vast amount of lifetime data of the participants, we could only measure knee breadth at one timepoint. We were thus limited to a cross-sectional design, without being able to address cause-effect relationships or longitudinal trends in knee dimensions. In addition, our MVPA measurements were collected at 46 years, and we did not have accelerometric data on PA from childhood or early adulthood. However, late adulthood activity appears to be in concordance with earlier activity [30]. Regarding potential confounders, comorbidities were not controlled for. As our analysis did not distinguish between leisure-time and occupational physical activity, their independent associations with knee breadth should be characterized in future studies. Studies are also welcomed to explore intensity-specific associations between physical activity and knee outcomes.

## Conclusion

In this population-based study, higher level of MVPA was associated with larger knee breadth among both women and men. Our cross-sectional findings suggest that articular surface size may differ according to activity level, thus contradicting previous studies. We speculate that higher MVPA may improve the robusticity of the bony knee structures and thus have an additional mechanism of action in knee problems. However, further studies are needed to confirm and investigate the association between articulation area and musculoskeletal health. In particular, longitudinal studies are needed to address causality.

## Supplementary Information

**Additional file 1.**

## Data Availability

The datasets generated and/or analysed during the current study are not publicly available due local privacy regulations but are available from the NFBC Project Center for applicants who meet criteria. Please see https://oulu.fi/nfbc/ for more information.

## References

[1] Gunter KB, Almstedt HC, Janz KF (2012). Physical activity in childhood may be the key to optimizing lifespan skeletal health. Exerc Sport Sci Rev.

[2] Nikander R, Sievänen H, Heinonen A, Daly RM, Uusi-Rasi K, Kannus P (2010). Targeted exercise against osteoporosis: A systematic review and meta-analysis for optimising bone strength throughout life. BMC Med.

[3] Nilsson M, Sundh D, Mellström D, Lorentzon M (2017). Current Physical Activity Is Independently Associated With Cortical Bone Size and Bone Strength in Elderly Swedish Women. J Bone Miner Res.

[4] Modarress-Sadeghi M, Oura P, Junno J-A, Niemelä M, Niinimäki J, Jämsä T (2019). Objectively Measured Physical Activity Is Associated with Vertebral Size in Midlife. Med Sci Sports Exerc.

[5] Haapasalo H, Kontulainen S, Sievänen H, Kannus P, Järvinen M, Vuori I (2000). Exercise-induced bone gain is due to enlargement in bone size without a change in volumetric bone density: a peripheral quantitative computed tomography study of the upper arms of male tennis players. Bone..

[6] Plochocki JH, Riscigno CJ, Garcia M (2006). Functional adaptation of the femoral head to voluntary exercise. Anat Rec A Discov Mol Cell Evol Biol.

[7] Frost HM (1999). Joint Anatomy, Design, and Arthroses: Insights of the Utah Paradigm. Anat Rec.

[8] Ruff CB, Scott WW, Liu AY (1991). Articular and diaphyseal remodeling of the proximal femur with changes in body mass in adults. Am J Phys Anthropol.

[9] Lieberman DE, Devlin MJ, Pearson OM (2001). Articular area responses to mechanical loading: effects of exercise, age, and skeletal location. Am J Phys Anthropol.

[10] Trinkaus E, Churchill SE, Ruff CB (1994). Postcranial robusticity in Homo. II: Humeral bilateral asymmetry and bone plasticity. Am J Phys Anthropol.

[11] Cain KL, Sallis JF, Conway TL, Van Dyck D, Calhoon L (2013). Using accelerometers in youth physical activity studies: a review of methods. J Phys Act Health.

[12] Slootmaker SM, Schuit AJ, Chinapaw MJ, Seidell JC, van Mechelen W (2009). Disagreement in physical activity assessed by accelerometer and self-report in subgroups of age, gender, education and weight status. Int J Behav Nutr Phys Act.

[13] Nordström T, Miettunen J, Auvinen J, Ala-Mursula L, Keinänen-Kiukaanniemi S, Veijola J, et al. Cohort Profile: 46 years of follow-up of the Northern Finland Birth Cohort 1966 (NFBC1966). Int J Epidemiol. 2021. 10.1093/ije/dyab109. 10.1093/ije/dyab109 PMC8743124 34999878

[14] University of Oulu. University of Oulu: Northern Finland Birth Cohort 1966. 1966. http://urn.fi/urn:nbn:fi:att:bc1e5408-980e-4a62-b899-43bec3755243. Accessed 14 Dec 2021.

[15] Niemelä MS, Kangas M, Ahola RJ, Auvinen JP, Leinonen A-M, Tammelin TH (2019). Dose-response relation of self-reported and accelerometer-measured physical activity to perceived health in middle age-the Northern Finland Birth Cohort 1966 Study. BMC Public Health.

[16] Kiviniemi AM, Perkiömäki N, Auvinen J, Niemelä M, Tammelin T, Puukka K (2017). Fitness, Fatness, Physical Activity, and Autonomic Function in Midlife. Med Sci Sports Exerc.

[17] Kinnunen H, Häkkinen K, Schumann M, Karavirta L, Westerterp KR, Kyröläinen H (2019). Training-induced changes in daily energy expenditure: Methodological evaluation using wrist-worn accelerometer, heart rate monitor, and doubly labeled water technique. PLoS One.

[18] Brugniaux JV, Niva A, Pulkkinen I, Laukkanen RMT, Richalet J-P, Pichon AP (2010). Polar Activity Watch 200: a new device to accurately assess energy expenditure. Br J Sports Med.

[19] Jauho A-M, Pyky R, Ahola R, Kangas M, Virtanen P, Korpelainen R (2015). Effect of wrist-worn activity monitor feedback on physical activity behavior: A randomized controlled trial in Finnish young men. Prev Med Rep.

[20] Department of Health Physical Activity Health Improvement and Protection (2011). Start active, stay active: a report on physical activity for health from the four home Countries’ chief medical officers.

[21] US Department of Health and Human Services (2008). 2008 physical activity guidelines for American.

[22] Tremblay MS, Warburton DER, Janssen I, Paterson DH, Latimer AE, Rhodes RE (2011). New Canadian physical activity guidelines. Appl Physiol Nutr Metab.

[23] World Health Organization (2010). Global recommendations on physical activity for health.

[24] Leinonen A-M, Ahola R, Kulmala J, Hakonen H, Vähä-Ypyä H, Herzig K-H (2017). Measuring physical activity in free-living conditions-comparison of three accelerometry-based methods. Front Physiol.

[25] Keisu A, Oura P, Niskanen M, Ruff CB, Niinimäki J, Arvola T (2019). The association between knee breadth and body mass: The Northern Finland Birth Cohort 1966 case study. Am J Phys Anthropol.

[26] Kan H, Arai Y, Kobayashi M, Nakagawa S, Inoue H, Hino M (2017). Radiographic Measurement of Joint Space Width Using the Fixed Flexion View in 1,102 Knees of Japanese Patients with Osteoarthritis in Comparison with the Standing Extended View. Knee Surg Relat Res.

[27] Niinimäki T, Ojala R, Niinimäki J, Leppilahti J (2010). The standing fixed flexion view detects narrowing of the joint space better than the standing extended view in patients with moderate osteoarthritis of the knee. Acta Orthop.

[28] Nieves JW, Formica C, Ruffing J, Zion M, Garrett P, Lindsay R (2005). Males have larger skeletal size and bone mass than females, despite comparable body size. J Bone Miner Res.

[29] Oura P (2017). Search for lifetime determinants of midlife vertebral size: emphasis on lifetime physical activity and early-life physical growth. Acta Univ Oul D 1418.

[30] Lounassalo I, Salin K, Kankaanpaä A, Hirvensalo M, Palomäki S, Tolvanen A (2019). Distinct trajectories of physical activity and related factors during the life course in the general population: A systematic review. BMC Public Health.

