# Supplementary material for: Accelerometer-measured physical activity is associated with knee breadth in middle-aged Finns – a population-based study
Source: BMC Musculoskelet Disord. 2022 May 31;23:517. doi: 10.1186/s12891-022-05475-7 (PMC9153128; doi:10.1186/s12891-022-05475-7)
Supplement: Supplementary file 1 — Additional file 1. [file 12891_2022_5475_MOESM1_ESM.docx]

**SUPPLEMENTARY MATERIAL**

**Supplementary Table 1**. Comparison between the present sample and those excluded.

| Characteristic | Sample  (n = 1508) | Excluded^1^  (n = 3979—5639) | P value |
| --- | --- | --- | --- |
| Sex |  |  |  |
| Female, % (n) | 45.2 (681) | 46.4 (2618) |  |
| Male, % (n) | 54.8 (827) | 53.6 (3021) | 0.380^2^ |
| Body mass index (kg/m^2^), mean (SD) | 26.2 (4.3) | 26.8 (4.9) | **< 0.001^3^** |
| Smoking |  |  |  |
| Non-smoker, % (n) | 54.6 (824) | 50.6 (2589) |  |
| Former smoker, % (n) | 28.4 (429) | 26.2 (1341) |  |
| Current smoker, % (n) | 16.9 (255) | 23.2 (1188) | **< 0.001^3^** |
| Education years |  |  |  |
| <9, % (n) | 3.2 (48) | 4.3 (221) |  |
| 9—12, % (n) | 69.6 (1050) | 68.9 (3577) |  |
| >12, % (n) | 27.2 (410) | 26.8 (1392) | 0.173^2^ |
| Knee breadth dimensions |  |  |  |
| Femoral biepicondylar breadth (mm), mean (SD) | 82.4 (6.3) | - | - |
| Femoral condylar mediolateral breadth (mm), mean (SD) | 76.5 (6.3) | - | - |
| Tibial plateau mediolateral breadth (mm), mean (SD) | 74.6 (5.8) | - | - |
| Accelerometer data |  |  |  |
| Moderate-to-vigorous physical activity (minutes/day), median (IQR) | 63 (45—86) | 63 (45—86) | 0.972^4^ |
| Accelerometer wear-time (minutes/day), median (IQR) | 977 (937—1014) | 984 (946—1019) | **< 0.001^4^** |

IQR = Interquartile range, SD = Standard deviation.

^1^N varies due to missing data.
^2^Chi square test.
^3^Independent-samples T test.

^4^Mann-Whitney U test.

**Supplementary Table 2**. Unadjusted, intermediate, and fully adjusted general linear models for the association between moderate-to-vigorous physical activity and knee breadth.

| Outcome and model | Women | |  | Men | |
| --- | --- | --- | --- | --- | --- |
|  | Beta^1^ (95% CI) | P value |  | Beta^1^ (95% CI) | P value |
| Femoral biepicondylar breadth (mm) | | |  |  |  |
| Unadjusted model | 1.00 (0.52—1.48) | **<0.001** |  | 0.99 (0.51—1.46) | **<0.001** |
| Intermediate model 1^2^ | 1.29 (0.81—1.78) | **<0.001** |  | 1.13 (0.66—1.61) | **<0.001** |
| Intermediate model 2^3^ | 1.30 (0.82—1.79) | **<0.001** |  | 1.19 (0.71—1.68) | **<0.001** |
| Intermediate model 3^4^ | 1.35 (0.86—1.83) | **<0.001** |  | 1.24 (0.76—1.73) | **<0.001** |
| Fully adjusted model^5^ | 1.42 (0.92—1.92) | **<0.001** |  | 1.27 (0.78—1.76) | **<0.001** |
| Femoral condylar mediolateral breadth (mm) | |  |  |  |  |
| Unadjusted model | 1.16 (0.72—1.61) | **<0.001** |  | 1.06 (0.61—1.50) | **<0.001** |
| Intermediate model 1^2^ | 1.30 (0.85—1.75) | **<0.001** |  | 1.17 (0.72—1.61) | **<0.001** |
| Intermediate model 2^3^ | 1.31 (0.85—1.76) | **<0.001** |  | 1.20 (0.75—1.65) | **<0.001** |
| Intermediate model 3^4^ | 1.34 (0.88—1.79) | **<0.001** |  | 1.25 (0.80—1.71) | **<0.001** |
| Fully adjusted model^5^ | 1.40 (0.94—1.86) | **<0.001** |  | 1.28 (0.82—1.73) | **<0.001** |
| Tibial plateau mediolateral breadth (mm) | |  |  |  |  |
| Unadjusted model | 1.01 (0.60—1.43) | **<0.001** |  | 1.03 (0.61—1.44) | **<0.001** |
| Intermediate model 1^2^ | 1.22 (0.80—1.64) | **<0.001** |  | 1.15 (0.73—1.57) | **<0.001** |
| Intermediate model 2^3^ | 1.21 (0.79—1.63) | **<0.001** |  | 1.17 (0.75—1.60) | **<0.001** |
| Intermediate model 3^4^ | 1.25 (0.83—1.67) | **<0.001** |  | 1.22 (0.79—1.65) | **<0.001** |
| Fully adjusted model^5^ | 1.26 (0.83—1.69) | **<0.001** |  | 1.21 (0.78—1.65) | **<0.001** |

CI = Confidence interval.
^1^Beta coefficients are interpreted as the contribution of 60 minutes/day of moderate-to-vigorous physical activity to the respective knee breadth measurement in mm.
^2^Adjusted for body mass index.

^3^Adjusted for body mass index and smoking.

^4^Adjusted for body mass index, smoking and education years.

^5^Adjusted for body mass index, smoking, education years and accelerometer weartime.

**
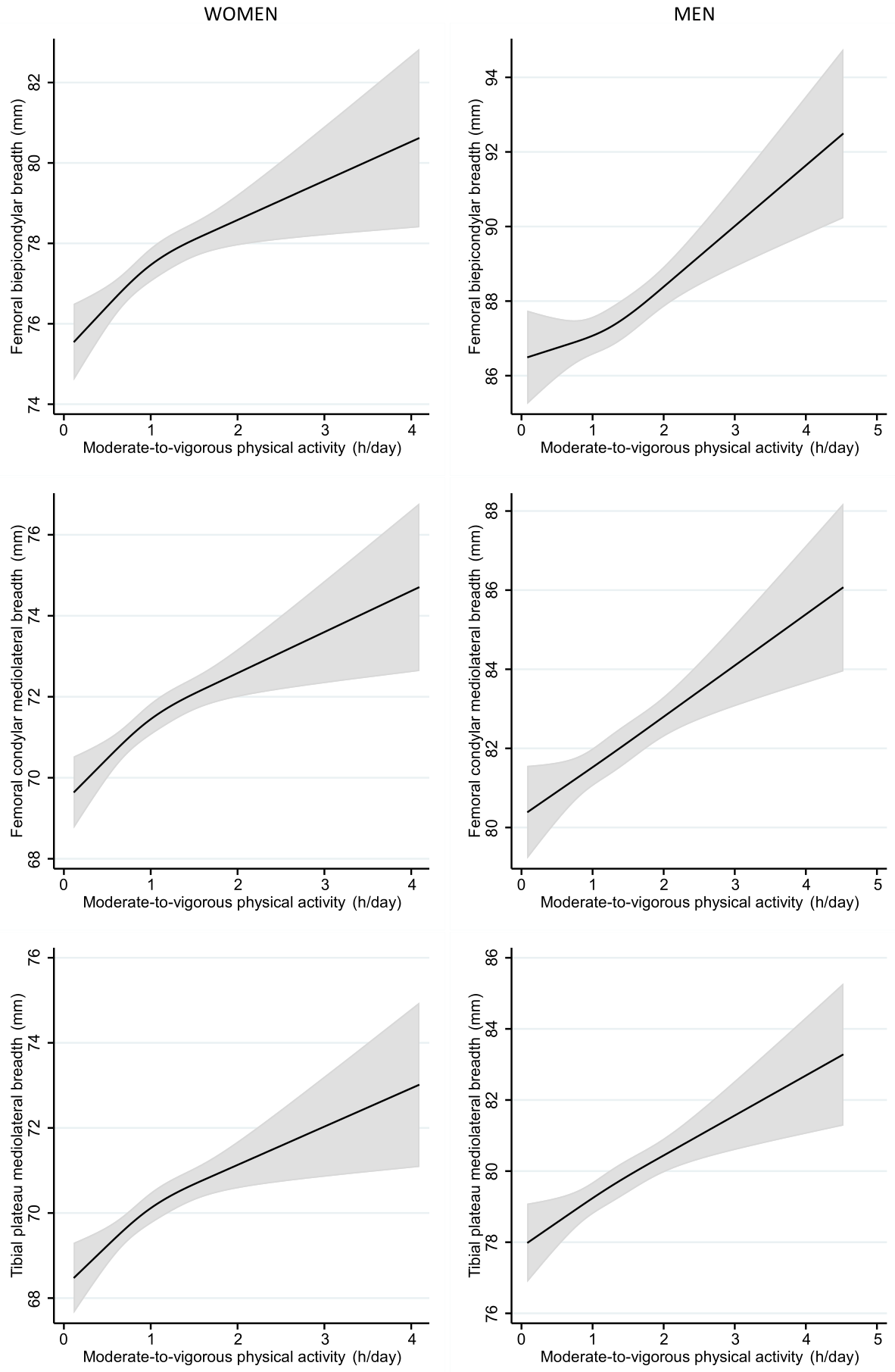
**

**Supplementary Figure 1**. Association between moderate-to-vigorous physical activity and knee breadth according to restricted cubic spline regression models.
